# *PLoS Computational Biology* Conference Postcard from PSB 2011

**DOI:** 10.1371/journal.pcbi.1002019

**Published:** 2011-03-31

**Authors:** A. Murat Eren

**Affiliations:** Department of Computer Science, University of New Orleans, New Orleans, Louisiana, United States of America; University of California San Diego, United States of America

We were pleased to see PLoS Conference Postcards return to the Pacific Symposium on Biocomputing (PSB), held January 4–11, 2011. This year we received a Postcard from A. Murat Eren, a PhD student at the University of New Orleans, in which he discusses a software package designed for use in microbial ecology research. We hope to see Conference Postcards at ISMB 2011, and if you would like to contribute a Postcard you can find out more at: http://www.ploscompbiol.org/doi/pcbi.1000746.

## John Bunge on “Estimating the Number of Species with CatchAll” in the Microbiome Studies Session

### Reported by A. Murat Eren, University of New Orleans

Microbial ecology, the relatively young and flourishing juncture of ecology and microbiology, had its own session at the Pacific Symposium on Biocomputing 2011 for the first time: “Microbiome Studies: Understanding How the Dominant Form of Life Affects Us”. This session hosted an introductory tutorial, paper presentations, and a prolific discussion session.

Microbial beings predominate life on this planet both in terms of abundance and diversity. The recent developments in massively parallel high-throughput sequencing technologies made 16S ribosomal RNA gene tag-based relative abundance and phylogenetic studies feasible, and this, in turn, helped scientists more deeply explore the diversity of microbial communities. With a greater understanding of the dynamics of these communities, we eventually will better explain reciprocal and correlative interactions between them and their environments.

Nevertheless, assessment of microbial diversity in a given environment is a cumbersome task as it confronts researchers with a fundamental problem: sampling bias. Mostly due to the vast scaling differences involved with sampling, reliable and applicable solutions to measure how well a sample represents a community's true diversity are almost impossible to develop. However, microbial ecologists still have to rely on their samples to speculate about the diversity of their original communities, and this requires heavy use of computational statistics and bioinformatics.

There are several widely used non-parametric and computationally lightweight diversity estimators that rely on abundance data, such as Chao1 and ACE, but they are known to be prone to skewed results when working with very high diversity situations where rare members create a long tail in the frequency count distribution curve of a sample. To address one of the major requisites of microbial ecology, biologists need more sophisticated and still computationally efficient quantitative approaches that can provide better accuracy on long-tailed microbial samples to estimate the diversity of their originating community.

That is why I believe the method and the software package that was presented in PSB 2011 by John Bunge, “Estimating the Number of Species with CatchAll”, was an exciting development.

CatchAll is a software package that aims to find the optimal finite-mixture of models with the best parameters in order to realistically explain the distribution of operational taxonomic units in a sample, so that the actual diversity of the parent population could be computed by extrapolating the final estimation. The result of the analysis with CatchAll is a list of estimation recommendations along with confidence intervals, goodness-of-fit estimations, and standard errors for researchers to investigate and select. What makes CatchAll promising is the fact that it is the first application to carry out parametric species richness estimation by efficiently combining statistics with heuristics, rather than only using a single coverage-based nonparametric richness estimation method for approximation.

In his presentation, Bunge benchmarked the performance of CatchAll with a large data set from The International Census of Marine Microbes (ICoMM, http://icomm.mbl.edu/) and showed encouraging results. When I asked David Mark Welch from Josephine Bay Paul Center of Marine Biological Laboratory about CatchAll, he said it is already being used by people writing up ICoMM summaries and it is going to be a part of VAMPS (http://vamps.mbl.edu/) very soon.

CatchAll can be downloaded from http://www.northeastern.edu/catchall/ and run in all mainstream operating system environments. It also is a part of MOTHUR (http://www.mothur.org/), and soon will be available within QIIME (http://qiime.sourceforge.net/).

The epilogue of Bunge's presentation was one of the possible future directions of microbial ecology research put into words: how are we going to incorporate the estimation of *unseen* diversity into analyses of *identities* of organisms across populations? All statistical methods (including CatchAll) will, at best, let us study our samples and estimate how much diversity we are missing. However, as Bunge pointed out in his talk, estimating “how many more there are” is only the first step. Estimating “who” might be there and guessing “who” might be missing from our samples would definitely be a game changer and is a challenge to both statisticians and computer scientists.

The discussion session of Microbiome Studies took place after the paper presentations and was directed by James A. Foster from the University of Idaho. Microbial ecologists, including invited speaker Rob Knight from the University of Colorado, Jack Gilbert from Argonne National Laboratory, and Thomas G. Doak from Indiana University, not only answered questions from scientists in other fields, but also discussed and listed issues of microbial ecology that are in need of attention.

During the discussion session two major challenges involved in diversity assessment efforts were portrayed: (1) sequencing errors that are introduced by sequencing methods and (2) the difficulty of separating noise from the actual rare members of an underlying population. These hurdles undoubtedly lead to mere approximations of the diversity in an environment instead of a factual representation of it. It was noted that even though CatchAll substantially improves the accuracy of the statistical robustness of the diversity estimation process, caveats introduced by the limits of 16S rRNA gene and today's high-throughput sequencing methods should always be considered.

One general suggestion that emerged from this discussion session was to focus on the functional role of the tail that represents rare individuals in microbial communities. It is intuitive to focus on dominant members of assemblages, but rare members might have an unanticipated impact on the *functional diversity* of their communities.

Another consensus emerging from the discussion session was the importance of defining higher order interactions of microbial populations with their human hosts. A vast amount of sequence data and meta-information is available as accessible online repositories. This allows researchers to develop and test hypotheses on minimum core sets of microbes that define diseases. Modeling the compositional complexity of microbial populations will definitely demand a serious amount of effort and time. Nevertheless, acknowledging this necessity and inviting computer scientists and statisticians to solve this puzzle might be the first step.

All conference materials and an online copy of the proceedings can be obtained from http://psb.stanford.edu/.

